# Prophylactic vs. Therapeutic Treatment With P2Et Polyphenol-Rich Extract Has Opposite Effects on Tumor Growth

**DOI:** 10.3389/fonc.2018.00356

**Published:** 2018-08-30

**Authors:** Paola Lasso, Alejandra Gomez-Cadena, Claudia Urueña, Alena Donda, Amaia Martinez-Usatorre, Alfonso Barreto, Pedro Romero, Susana Fiorentino

**Affiliations:** ^1^Grupo de Inmunobiología y Biología Celular, Pontificia Universidad Javeriana, Bogotá, Colombia; ^2^Department of Oncology, Faculty of Biology and Medicine, University of Lausanne, Lausanne, Switzerland

**Keywords:** polyphenols, immune response, cancer, immunomodulation, proinflammatory

## Abstract

Polyphenols have tumoricidal effects via anti-proliferative, anti-angiogenic and cytotoxic mechanisms and have recently been demonstrated to modulate the immune response through their anti- or pro- oxidant activity. Nevertheless, it remains controversial whether antioxidant-rich supplements have real beneficial effects on health, especially in complex diseases such as cancer. We previously identified a polyphenol-rich extract obtained from *Caesalpinia spinosa* (P2Et) with anti-tumor activity in both breast carcinoma and melanoma. The present work evaluated the ability of P2Et extract to modulate the immune system in either the steady state or following tumor challenge. We found that the prophylactic treatment of healthy mice increased the number of CD4^+^ and CD8^+^ activated T, NK, regulatory T, dendritic and myeloid-derived suppressor cells in lymphoid organs together with a significant increase in plasma IL-6. Interestingly, this pre-conditioning of the host immune system with P2Et did not involve a protective effect against the control of tumor growth and metastasis in transplantable models of melanoma (B16) and breast cancer (4T1), but in contrast, a detrimental effect was observed in both models. We further demonstrated that this effect was at least partly due to an increase in regulatory T cells, myeloid-derived suppressor cells, and proinflammatory cytokines, with a concomitant decrease in CD4^+^ and CD8^+^ T cells. Taken together, these results suggest that the anti-tumor and immunomodulation properties of the P2Et extract critically depend on the presence of the tumor and might be mediated by the complex interactions between the tumor cells and the other components of the tumor microenvironment.

## Introduction

Cancer is a major public health problem and remains one of the major causes of mortality and morbidity worldwide despite major advances in treatment. In addition to surgery, cancer therapy includes chemotherapy and radiotherapy, which may cause severe off-target toxicity and are not equally effective in all patients. The identification of the immune system as a key player in the control of tumor growth and progression recently led to the development of different immunotherapy strategies, such as cancer vaccines, immune modulator antibodies and adoptive cell transfer therapy (1). Despite resounding clinical successes, immunotherapy has shown durable clinical benefit only in a certain proportion of patients. Consequently, a search for alternative treatments or combined strategies against cancer remains necessary (2).

Natural products, which are acquiring increasing importance, can prevent tumor development due to their antioxidant, anti-proliferative, anti-angiogenic and immune modulator properties (3–5). Nevertheless, the health benefits of consuming antioxidant supplements remain controversial since some studies have shown advantages (6–9) but others consider them detrimental (10–13). In this context, supplements with vitamin E and N-acetylcysteine (NAC) have been shown to lead to greater progression of lung cancer (14) and increased melanoma metastasis formation (15). Previously, our group identified a gallotannin-rich extract from *Caesalpinia spinosa* (P2Et) as an anti-tumor agent in breast and melanoma murine models (16–18). This extract contains high proportions of gallic acid-containing compounds (galloylquinic acids and glucosyl-gallate compounds), shows important antioxidant activity and is highly and specifically cytotoxic against tumor cells, in particular those expressing drug resistance pumps such as Pgp (19). Furthermore, the anti-tumor activity of P2Et requires an intact adaptive immune system in the B16F10 melanoma model (16).

In this study, we assessed the capacity of the P2Et extract to modulate the immune system in either the steady state or following tumor challenge. We observed that P2Et indeed has immune modulatory properties when administered to healthy mice. However, in contrast to its anti-tumor activity in tumor-bearing mice, prophylactic treatment with P2Et led to enhanced tumor growth of engrafted breast and melanoma tumors. This effect was at least partly due to the increased production of IL-6. Taken together, these results suggest that the anti-tumor activity and immune modulation properties of the P2Et extract critically depend on the presence of the tumor and might be mediated by the complex interactions between tumor cells and other components of the tumor microenvironment.

## Materials and methods

### Plant material

*Caesalpinia spinosa* pods were collected in Villa de Leyva, Boyacá, Colombia and identified by Luis Carlos Jiménez from the Colombian National Herbarium (voucher specimen number COL 523714, Colombian Environmental Ministry agreement number 0454 related to the use of genetic resources and derived products). The P2Et extract was produced under GMP conditions and chemically characterized as previously described (19, 20).

### Mice

Young (6–12 weeks old) female C57BL/6 and BALB/c mice were purchased from the Jackson Laboratories (Bar Harbor, ME, USA) and housed at the animal facilities of the Pontificia Universidad Javeriana (PUJ, Bogotá, Colombia) following the established protocols of the Ethics Committee of the Faculty of Sciences, PUJ, and National and International Legislation for Live Animal Experimentation (Colombia Republic, Resolution 08430, 1993; National Academy of Sciences, 2010). The present study was approved by the ethics committee of the Faculty of Sciences, PUJ, on May 6, 2012. Each specific protocol was also approved by the animal experimentation committee of PUJ. Mice were maintained in polyethylene cages with food and water provided *ad libitum*, on a 12-h light/dark cycle at 20–22°C and 40–60% humidity. The Tyr::NRas mice were breed with INK4a knockout mice to obtain the Tyr-NRas^Q61k^::Cdk4^R24C^ spontaneous mouse melanoma model. The breeding and experiments with Tyr-NRas^Q61k^::Cdk4^R24C^ mice were performed at the animal facility (Epalinges) of the University of Lausanne where the colonies are established. This model was tested under the approval of the Veterinary authority of Canton of Vaud, Switzerland and under authorization VD1850. Experiments were performed in accordance with Swiss ethical guidelines.

### Tumor cell lines and culture conditions

The murine melanoma B16F10 cell line was kindly provided by PR (Ludwig Center for Cancer Research, Department of Oncology—Faculty of Biology and Medicine University of Lausanne, Switzerland). The B16 mouse model was created in the 1970s from a melanoma that developed spontaneously in the ear of a female C57BL/6 mouse and was then passaged *in vivo* to create the B16F10 line (21). The murine breast cancer 4T1 cell line was grown in BALB/c mice and developed into a highly tumorigenic and invasive tumor that can spontaneously metastasize from a primary tumor in the mammary gland to multiple distant sites (22). The 4T1 mammary tumor cells were kindly provided by Dr Alexzander Asea (Texas A&M Health Science Center College of Medicine, Temple, TX). Cells were cultured in RPMI-1640 (Eurobio, Toulouse, France) supplemented with 10% heat-inactivated fetal bovine serum (FBS) (Eurobio), 2 mM L-glutamine, 100 U/ml penicillin, 100 μg/ml streptomycin, 0.01 M HEPES buffer and 1 mM sodium pyruvate (Eurobio) and incubated in a humidified environment at 37°C and 5% CO_2_. Cells were grown until 75% confluency and passaged using trypsin/1X EDTA (Eurobio), washed with PBS and resuspended in supplemented RPMI-1640.

### Abs

The following Abs were used for cell surface staining: anti-CD3 Pacific Blue (clone 17A2), anti-CD8 PE Texas Red (clone 53.6.7), anti-CD44 PE-Cy7 (clone IM7), anti-B220 PE Texas Red (clone RA3-6B2), anti-NK1.1 FITC (clone PK136), anti-CD69 Alexa Fluor 647 (clone H1-2F3), anti-Ly6C APC-Cy7 (AL-21), anti-Ly6G PE-Cy7 (clone 1A8) (Biolegend, San Diego, CA, USA), anti-CD4 APC e-Fluor 780 (clone GK1.5), anti-CD62L PE-Cy5 (clone MEL-14) (eBiosciences, San Diego, CA, USA), anti-CD45RB PE (clone 16A), anti-CD3 Alexa Fluor 647 (clone 17A2), anti-CD4 PerCP (clone RM4-5), anti-CD8 PE (clone 53-6.7), anti-CD45 PE-Cy5 (clone 30-F11), anti-CD45 PE (clone 30-F11), anti-CD11c FITC (clone HL3) and anti-CD11b Alexa Fluor 700 (clone M1/70) (BD Biosciences, San José, CA, USA). The abs for intracellular staining included anti-FoxP3 Alexa Fluor 488 (clone MF23), anti-IFNγ Alexa Fluor 700 (clone XMG1.2), TNFα PE-Cy7 (clone MP6-XT22), IL-2 FITC (clone JES6-5H4) (BD Biosciences) and anti-CTLA-4 PE (clone UC10-4F10-11) (Biolegend). A LIVE/DEAD Fixable Aqua Dead Cell Stain Kit (Invitrogen Molecular Probes, Eugene, OR) was used for dead cell exclusion.

### *In vivo* tumor development experiments and treatment

**Figure 1:**
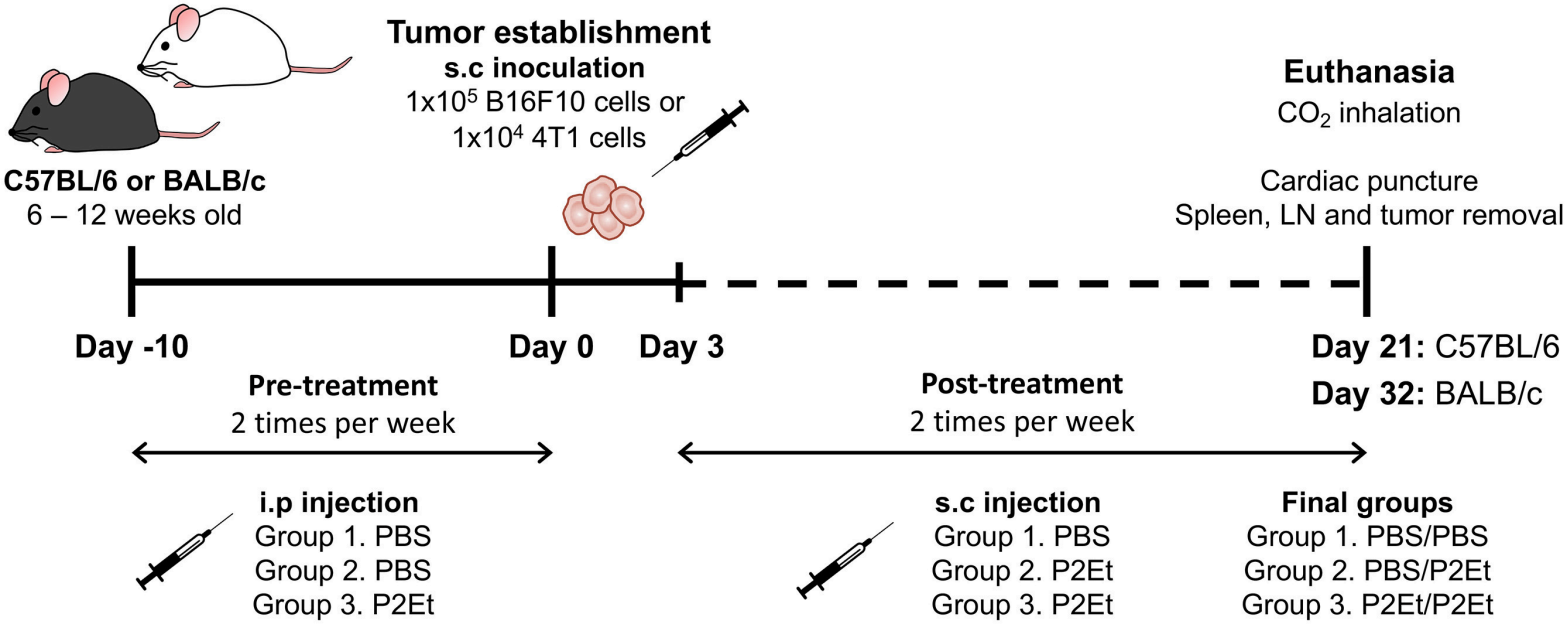
Experimental treatment scheme. To evaluate the effect of P2Et as prophylactic therapy in both murine models, mice received the following treatment scheme: PBS injection → tumor cells → PBS injection (PBS/PBS), PBS injection → tumor cells → P2Et treatment (PBS/P2Et), and P2Et treatment → tumor cells → P2Et treatment (P2Et/P2Et). The pretreatment was administered via i.p two times per week for 10 days, the tumor was later established by injection of B16F10 or 4T1 cells, and finally, 3 days after tumor cell injection, the post-treatment was s.c. administered around the tumor two times per week for 21 and 32 days, respectively.

For melanoma tumor induction, C57BL/6 mice were subcutaneously (s.c.) inoculated in the right flank with 1 × 10^5^ viable B16F10 cells suspended in 100 μl of PBS. For the breast cancer murine model, 1 × 10^4^ viable 4T1 cells suspended in 100 μl of PBS were s.c. injected into the right mammary fat pad of BALB/c mice. To evaluate the effect of P2Et treatment on tumor growth, 3 days after B16F10 inoculation, PBS (negative control) or P2Et extract (75 mg/kg body weight in 200 μl of PBS) were s.c. administered around the tumor two times per week. The P2Et therapeutic dose used was 4-fold lower than the LD-50 (median lethal dose) to ensure low toxicity. To determine whether P2Et modulates the immune response in healthy C57BL/6 or BALB/c mice, the animals were intraperitoneally (i.p.) injected with P2Et or PBS two times per week for 21 (C57BL/6) or 32 (BALB/c) days. To evaluate the effect of P2Et on tumor growth in a spontaneous melanoma model (23), Tyr-NRas^Q61k^::Cdk4^R24C^ mice were used (24). These mice were first synchronized for the appearance of melanoma tumors by topical treatment with the carcinogen DMBA on a shaved zone of the back, once per week for 5 weeks (100 μl of 0.5 mg/ml in acetone). Treatment was initiated at week 4 after birth. Tumors started to appear 9 weeks after the first DMBA treatment, and the mice started P2Et treatment (75 mg/kg) twice per week as soon as the tumors were visible until the end of the experiment. To evaluate the effect of P2Et as prophylactic therapy in both murine models, mice received the following treatment scheme: PBS injection → tumor cells → PBS injection (PBS/PBS), PBS injection → tumor cells → P2Et treatment (PBS/P2Et), and P2Et treatment → tumor cells → P2Et treatment (P2Et/P2Et). In all cases, the pretreatment was i.p administered two times per week for 10 days, followed by the injection of B16F10 or 4T1 cells, and finally, 3 days after tumor cell injection, the post-treatment was s.c. administered around the tumor two times per week for 21 and 32 days, respectively (Figure 1). In all experimental settings, the size of the tumors was assessed three times per week with Vernier calipers, and the volume was calculated according to the formula V (mm^3^) = L (major axis) × W^2^ (minor axis)/2 (25). Mice were euthanized by CO_2_ inhalation, and then spleen, draining inguinal lymph nodes (LN), and tumor were removed. Tumor weight was determined using a high-sensitivity balance.

### Flow cytometry and intracellular cytokine staining assays

Single cell suspensions were obtained from spleen, LN and tumor by mechanical or enzymatic dissociation. Briefly, 1 × 10^6^ cells were stained with LIVE/DEAD Fixable Aqua for 20 min in the dark at room temperature. After washing with PBS 2% FBS, the cells were stained for 30 min at 4°C in the dark with surface antibodies according to the designed multicolor panels. Then, the cells were washed and resuspended in 300 μl of PBS 2% FBS. To identify regulatory T cells, cells previously marked with anti-CD4 and anti-CD3 were fixed, permeabilized and stained with anti-FoxP3 and anti-CTLA-4 antibodies for 30 min at room temperature in the dark. The cells were then washed and resuspended in 300 μl of PBS 2% FBS.

To evaluate the functional activity of spleen cells from healthy mice after P2Et treatment, 2 × 10^6^ splenocytes were cultured with 3 μg/mL of P2Et extract or EtOH as a control for 24 h at 37°C and 5% CO_2_. The cells were then cultured with phorbol 12-myristate 13-acetate (PMA) and ionomycin (P/I) or without a stimulus for 7 h. The last 6 h of culture were performed in the presence of brefeldin A (1 μg/ml) (BD Pharmingen). Briefly, the cells were stained with LIVE/DEAD Fixable Aqua Dead Cell Stain for 20 min in the dark at room temperature. After washing with PBS 2% FBS, the cells were stained for 30 min at 4°C in the dark with anti-CD3, anti-CD4, anti-CD8 and anti-CD45 antibodies. Later, the cells were washed, fixed and permeabilized and then stained with anti-IFNγ, TNFα and IL-2 for 30 min at 4°C in the dark. Finally, the cells were washed and resuspended in 300 μl of PBS 2% FBS. Cells were acquired by flow cytometry using a FACSAria II flow cytometer (BD Immunocytometry Systems, San José, CA, USA), and the results were subsequently analyzed using FlowJo 9.3.2 software (Tree star, Ashland, OR). Pie charts were represented using Pestle version 1.7 and SPICE version 6.0 software (the National Institutes of Health, Bethesda, MD) (26).

### Cytokine assay

Serum was prepared from blood obtained by cardiac puncture, and cytokine evaluation was performed using a Cytometric Bead Array (CBA) mouse Th1, Th2, Th17 cytokine kit (BD Biosciences) according to the manufacturer's instructions. Experiments were performed twice, and each experiment was performed in duplicate. Events were acquired using a FACSAria II flow cytometer (BD Immunocytometry Systems), and the results were subsequently analyzed using FCAP array software version 3.0 (BD Biosciences). Data were log-transformed and plotted as the mean ± SEM.

### Cell-based antioxidant activity assay

Splenocytes from C57BL/6 and BALB/c healthy mice were seeded at a density of 2 × 105/well on a 96-well microplate in 200 μl medium/well at 0, 1 or 10 μM H2O2 (Sigma). Two hours after treatment, the medium was removed, and the cells were incubated with EtOH, 10 μM or 100 μM Trolox (Sigma), and 3 or 30 μg/ml of P2Et. 12 or 24 h after treatments, medium was removed, and the cells were washed twice with 200 μl of 1X PBS. Then, the cells were incubated for 40 min at 37°C with 100 μl/well of 1 μM 7′-dichlorofluorescin diacetate (DCFH-DA) in Hank's Balanced Salt Solution (HBSS) buffer. Cells were washed with 100 μl of 1X PBS and resuspended in 200 μl of 1X PBS containing propidium iodide. Cells were acquired by flow cytometry using a FACSAria II flow cytometer (BD Immunocytometry Systems, San José, CA, USA), and the results were subsequently analyzed using FlowJo 9.3.2 software (Tree star, Ashland, OR).

### Statistical analysis

Statistical analysis of the significance between two groups was calculated using the Mann–Whitney *U* test. For all cases, the differences were considered statistically significant when *p* < 0.05. GraphPad Prism version 6.0 for Mac OS X statistics software (GraphPad Software, San Diego, CA) was used for the statistical analyses. The pie charts were compared using 10,000 permutations calculated with the software SPICE version 6.0.

## Results

### The anti-tumor effect of P2ET is lost when administered as prophylactic treatment

**Figure 2:**
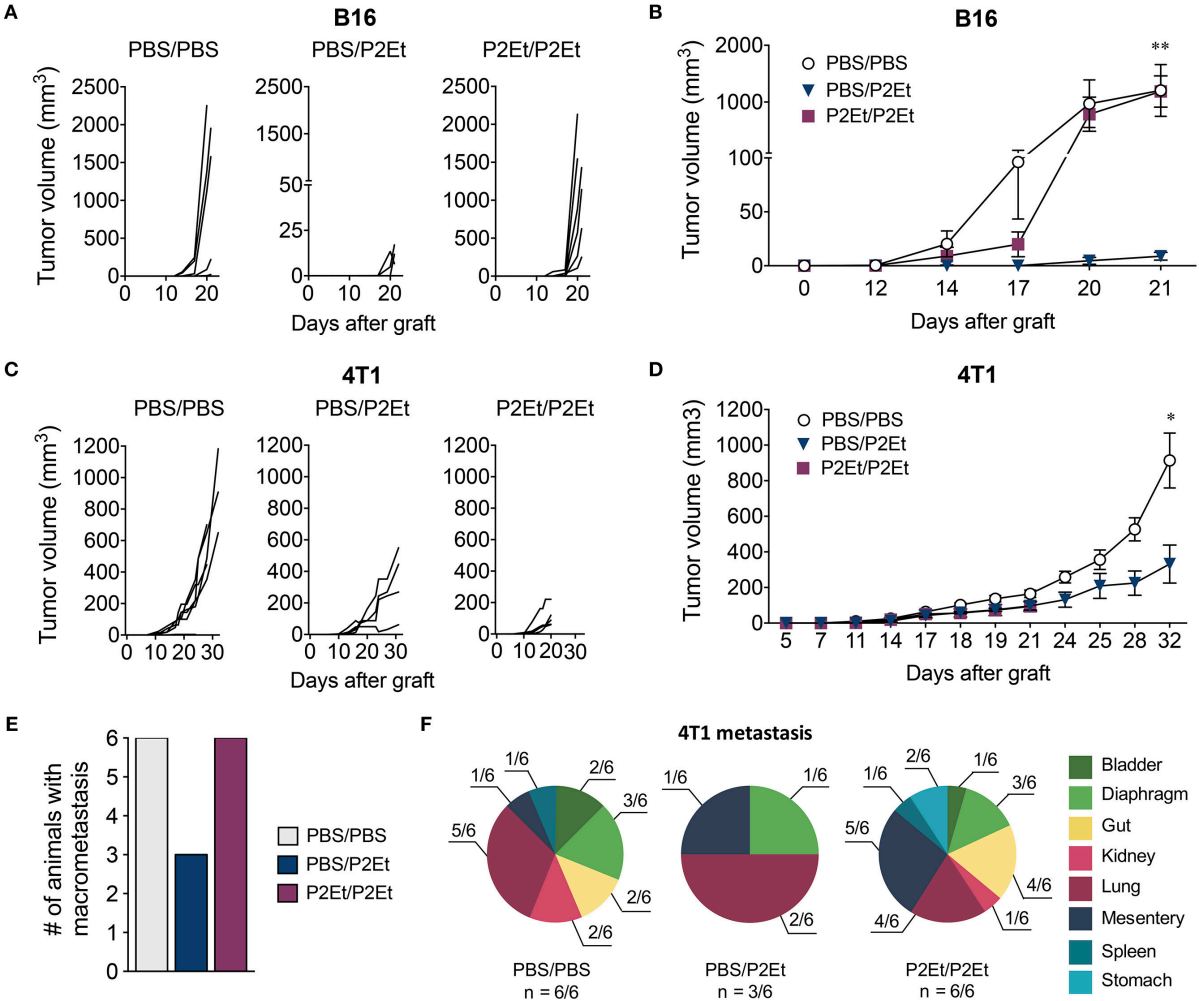
*In vivo* P2Et treatment delays melanoma and breast tumor growth, but this effect is lost when P2Et pretreatment is also provided. **(A)** B16F10 individual tumor volumes for all groups. **(B)** B16F10 tumor growth curve. **(C)** 4T1 individual tumor volumes for all groups. **(D)** 4T1 tumor growth curve. **(E)** 4T1 frequency of metastasis among the different groups. **(F)** Distribution of multi-organ metastasis of 4T1 tumors for all groups. *n* corresponds to the number of mice with metastases. In all cases, data are represented as the mean ± SEM. The *p* values were calculated using a Mann-Whitney *U* test. *p < 0.05, **p < 0.01.

We have previously described that P2Et treatment post-tumor engraftment delays both melanoma and breast cancer tumor growth (16–18). We wanted to assess whether P2Et as prophylactic treatment could prevent tumor growth. To answer to this question, we treated mice with P2Et or PBS (control) for 10 days 2 times per week, prior to engraftment of either 1 × 10^5^ B16F10 or 1 × 10^4^ 4T1 cells. Subsequently, P2Et or PBS administration s.c. in the periphery of the tumor was continued (Figure 1). In the melanoma B16 model, we confirmed that therapeutic treatment of established tumors with P2Et led to significantly delayed tumor growth compared with the other groups. Strikingly, however, this anti-tumor effect was lost when a prophylactic P2Et treatment was added, as shown by rapid tumor growth in all mice in the P2Et/P2Et group (Figures 2A,B). In the 4T1 breast cancer model, therapeutic treatment with P2Et also delayed tumor growth in PBS/P2Et mice (Figures 2C,D). Interestingly, the addition of prophylactic P2Et treatment showed a different pro-tumoral effect compared with B16 tumors. Indeed, the growth of the primary 4T1 tumors was slower than in the PBS group, but the frequency of metastasis was higher both in terms of incidence and number of organs invaded by 4T1 tumor cells (Figures 2E,F), which necessitated the euthanasia of these mice 12 days before the other groups (Figures 2C,D). Moreover, a higher proportion of both mice and organs were affected in the P2Et/P2Et treatment group compared with the PBS/PBS or PBS/P2Et groups at day 32 after grafting (Figures 2E,F). Indeed, only three mice from the PBS/P2Et group had visible metastases, and only 3 organs were affected (Figure 2F).

To evaluate the possibility that prophylactic treatment with the P2Et extract could directly act on T cells and inhibit their functional capacity, we treated spleen cells from C57BL/6 and BALB/c healthy mice with P2Et for 24 h, and cytokine production of T cells was assessed after PMA/ionomycin stimulation. We found that P2Et extract did not directly affect the functional capacity of T cells from C57BL/6 (Supplementary Figures 1A,B) or BALB/c (Supplementary Figures 1C,D). These results suggested that the unexpected findings regarding overgrowth or increased metastatic ability of tumor cells in P2Et/P2Et mice in both models might be due to indirect effects on other components of the tumor microenvironment. In this sense, we evaluate the effect of P2Et over the oxidative stress of splenocytes from healthy mice. For this, the cells were cultured for 2 h with medium (basal) or with H2O2 to induce oxidative stress. It was found that P2Et, like Trolox, has antioxidant activity in splenocytes from BALB/c (Supplementary Figures 2A,B) and C57BL/6 (Supplementary Figures 2C,D) healthy mice in basal and oxidative stress conditions. Additionally, this antioxidant effect was observed both at 12 (Supplementary Figures 2A,C) and 24 h (Supplementary Figures 2B,D), and it was dose depended. These results show that P2Et can protect normal splenocytes from oxidative aggression.

### Prophylactic P2ET-based therapy is associated with the generation of an immunosuppressive microenvironment that promotes tumor growth

**Figure 3:**
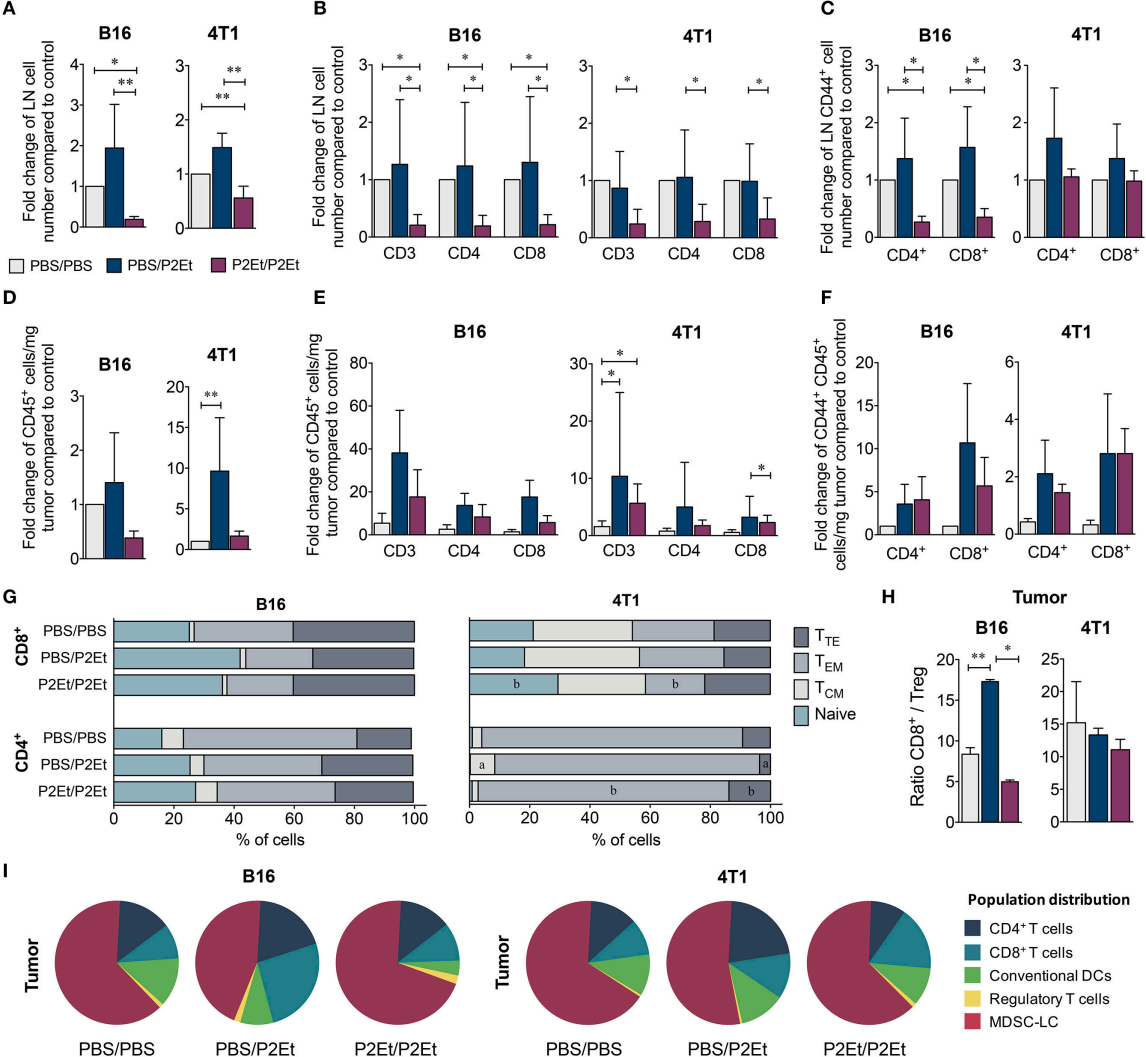
P2Et pretreatment decreases T cell recruitment into the tumor induced after P2Et post-engraftment therapy. Fold change in total LN cells numbers **(A)** in CD3^+^, CD4^+^, and CD8^+^ T cells **(B)** and in CD44^+^ activated T cells **(C)** in both tumor models and all groups. Fold change in CD45^+^ cell numbers per mg of tumor, **(D)** CD3 ^+^, CD4 ^+^, and CD8^+^ T cells **(E)**, and activated CD44^+^ T cells **(F)** in both models and all groups. Percentage of CD4^+^ and CD8^+^ T cells with naive, T_CM_, T_EM_, and T_TE_ phenotypes in tumors **(G)**. Tumor ratio of CD8^+^ T cells/Treg **(H)**. Pie charts of the distribution of tumor-infiltrating populations in each group **(I)**. In all cases, data are represented as the mean ± SEM. The *p* values were calculated using a Mann-Whitney *U* test. *p < 0.05, **p < 0.01.

To determine whether P2Et treatment pre- and post-engraftment induces changes in murine immune system reactivity, the frequency and distribution of different cell populations from spleen, lymph nodes (LN) and tumors of both murine models were evaluated comparing mice administered or not a prophylactic course of P2Et. Both in the B16F10 and 4T1 murine models, P2Et/P2Et mice had fewer CD45^+^ cells in the draining lymph nodes than the control group (Figure 3A). Likewise, this difference was also observed when we evaluated CD3^+^, CD4^+^, and CD8^+^ T cells in B16F10 and 4T1 LN of tumor-bearing mice (Figure 3B). We also found a reduced number of activated T cells (CD44^+^) in draining LN of B16F10 tumors, while no differences were observed in the 4T1 model, (Figure 3C). In the tumor, we observed an increase in CD45^+^ tumor-infiltrating cells in the PBS/P2Et group in both models (Figure 3D), which appeared to be CD3^+^, CD4^+^, or CD8^+^ T cells (Figure 3E) and were also more activated (Figure 3F), compared with the control group. Although the differences were not clear in terms of memory cell subpopulations from the tumor (Figure 3G), it was clear that P2Et/P2Et mice in 4T1 had a higher, but not significant, frequency of CD4^+^ naïve (T_N_, CD45RB^+^, CD62L^+^) T cells but a lower frequency of CD4^+^ effector memory T cells (T_EM_, CD45RB^−^, CD62L^−^) than the PBS/P2Et group (Figure 3G). Likewise, P2Et/P2Et mice had higher frequencies of naïve CD8^+^ T cells and lower frequencies of CD8^+^ T_EM_ cells (Figure 3G). In addition, the PBS/P2Et group had more CD8^+^ T cells than regulatory T cells in B16 tumors, which may favor the tumor control. This favorable CD8/Treg ratio was not observed in 4T1 tumors (Figure 3H), suggesting that the strain, as well as the type of tumor, may be at the origin of these differences. The assessment of other immune cell populations in the tumor showed that in both models, the P2Et/P2Et groups had increased proportions of tumor-infiltrating MDSC-LC (27) and a higher number of CD4^+^ and CD8^+^ T cells than the other groups (Figure 3I).

**Figure 4:**
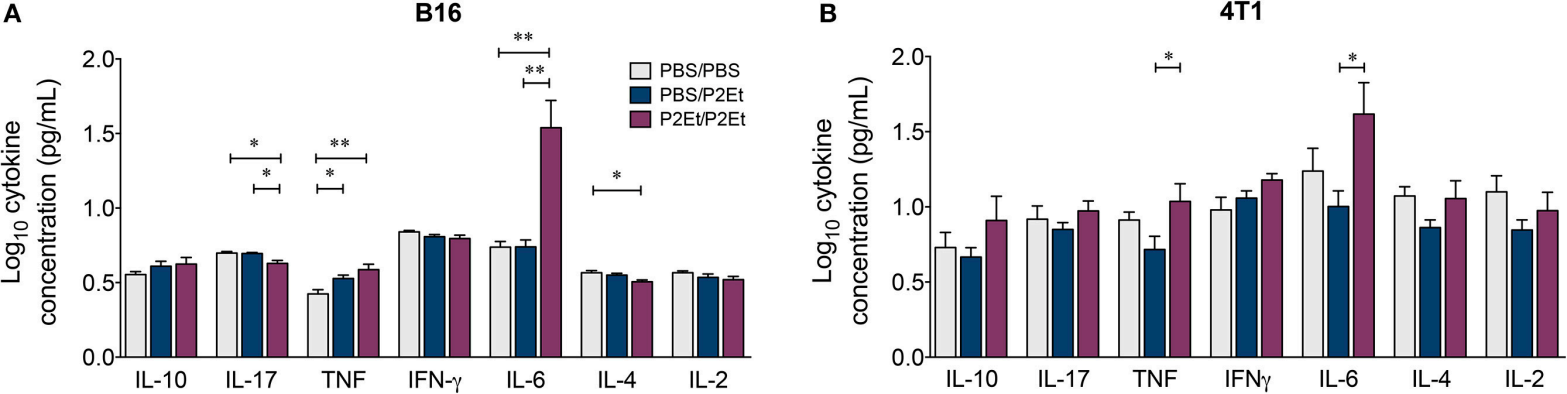
*In vivo* pre- and post-P2Et treatment favors the production of proinflammatory cytokines. Cytokine levels in the serum of B16 tumor-bearing mice **(A)** or 4T1 tumor-bearing mice **(B)** with PBS/PBS, PBS/P2Et or P2Et/P2Et treatment. The data were log-transformed and plotted as the mean ± SEM. The *p* values were calculated using a Mann-Whitney *U* test. *p < 0.05, **p < 0.01.

Interestingly, a significant increase in the serum concentrations of TNF and IL-6 was found in the P2Et/P2Et groups compared with the other groups in both tumor models (Figures 4A,B), while in the P2Et/P2Et-treated B16 tumors, the serum concentrations of both IL-17 and IL-4 were significantly decreased (Figure 4A).

### The P2ET extract from *Caesalpinia spinosa* modulates the immune response in healthy C57BL/6 and BALB/c mice

**Figure 5:**
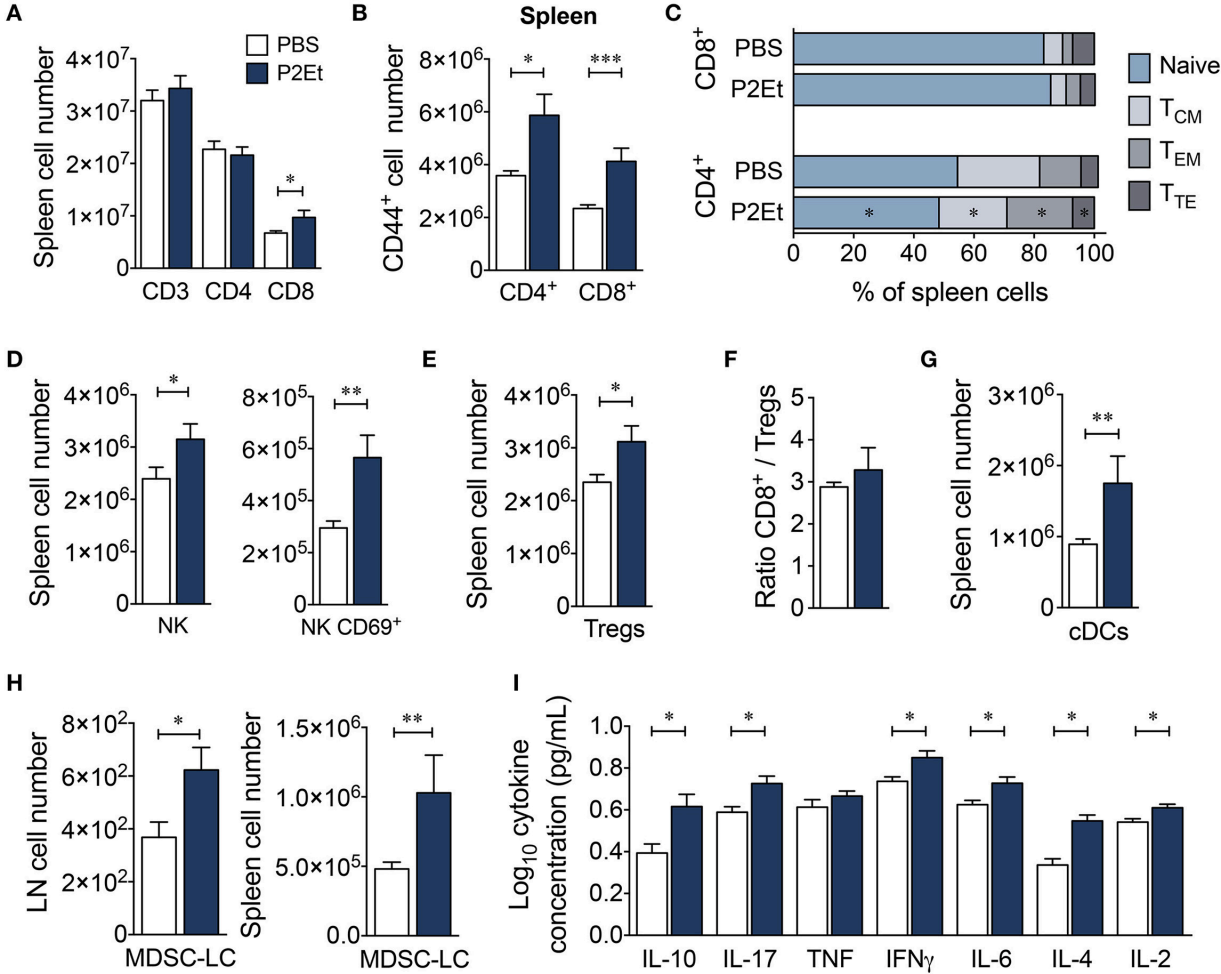
P2Et treatment modulates the immune response in healthy C57BL/6 mice. **(A)** Absolute numbers of CD3^+^, CD4^+^ and CD8^+^ T cells in spleen from healthy mice treated with P2Et or PBS (control). **(B)** Splenic CD4^+^ and CD8^+^ T cells expressing the CD44^+^ marker. **(C)** Percentages of the different populations (naive, T_CM_, T_EM_ and T_TE_) of splenic CD4^+^ and CD8^+^ T cells in mice with or without treatment. Absolute numbers of splenic NK and CD69-expressing NK cells **(D)** and regulatory T cells **(E)** in healthy mice treated with P2Et or PBS **(F)**. Ratio of splenic CD8^+^/Treg cells. **(G)** Conventional splenic DC numbers in each group. **(H)** Absolute numbers of MDSC-LCs in lymph nodes and spleen. **(I)** Cytokine concentration in serum of PBS or P2Et-treated healthy C57BL/6 mice measured by CBA. In all cases, the data are represented as the mean ± SEM. The *p* values were calculated using a Mann-Whitney *U* test. *p < 0.05, **p < 0.01, ***p < 0.001.

To ascertain whether the presence of a tumor was necessary for the effects of the P2Et extract on the immune system, we measured the serum levels of Th1/Th2/Th17 cytokines and assessed the distribution of several immune cell populations in healthy C57BL/6 mice treated with P2Et. Treatment with P2Et two times per week for 21 days increased the numbers of CD8^+^ T cells (Figure 5A) and the frequency of activated CD4^+^ and CD8^+^ T cells in the spleen compared with the control mice (Figure 5B). Moreover, mice treated with P2Et showed more CD4^+^ T_EM_ cells in spleen and LN and fewer T_N_ cells and central memory (T_CM_, CD45RB^−^, CD62L^+^) CD4^+^ T cells in spleen compared with the control group (Figure 5C). However, no differences were found in CD8^+^ T cells subsets (Figure 5C). Furthermore, we observed increased numbers of total NK and CD69^+^ activated NK cells (Figure 5D) along with more regulatory CTLA4^+^, Foxp3^+^ T cells (Treg) in the spleen of P2Et-treated mice (Figure 5E). However, the ratio of CD8^+^/Treg cells was the same in mice with or without treatment (Figure 5F), suggesting that P2Et might favor the mobilization of CD8^+^ cells to the spleen. Dendritic cells (DCs) were increased in the spleen of P2Et-treated mice (Figure 5G) as well as MDSC-LCs, which were increased in both spleen and LN (Figure 5H). Moreover, P2Et-treated mice had increased serum concentrations of IL-10, IL-17, IFN-γ, IL-6, IL-4, and IL-2 compared with the control group (Figure 5I).

**Figure 6:**
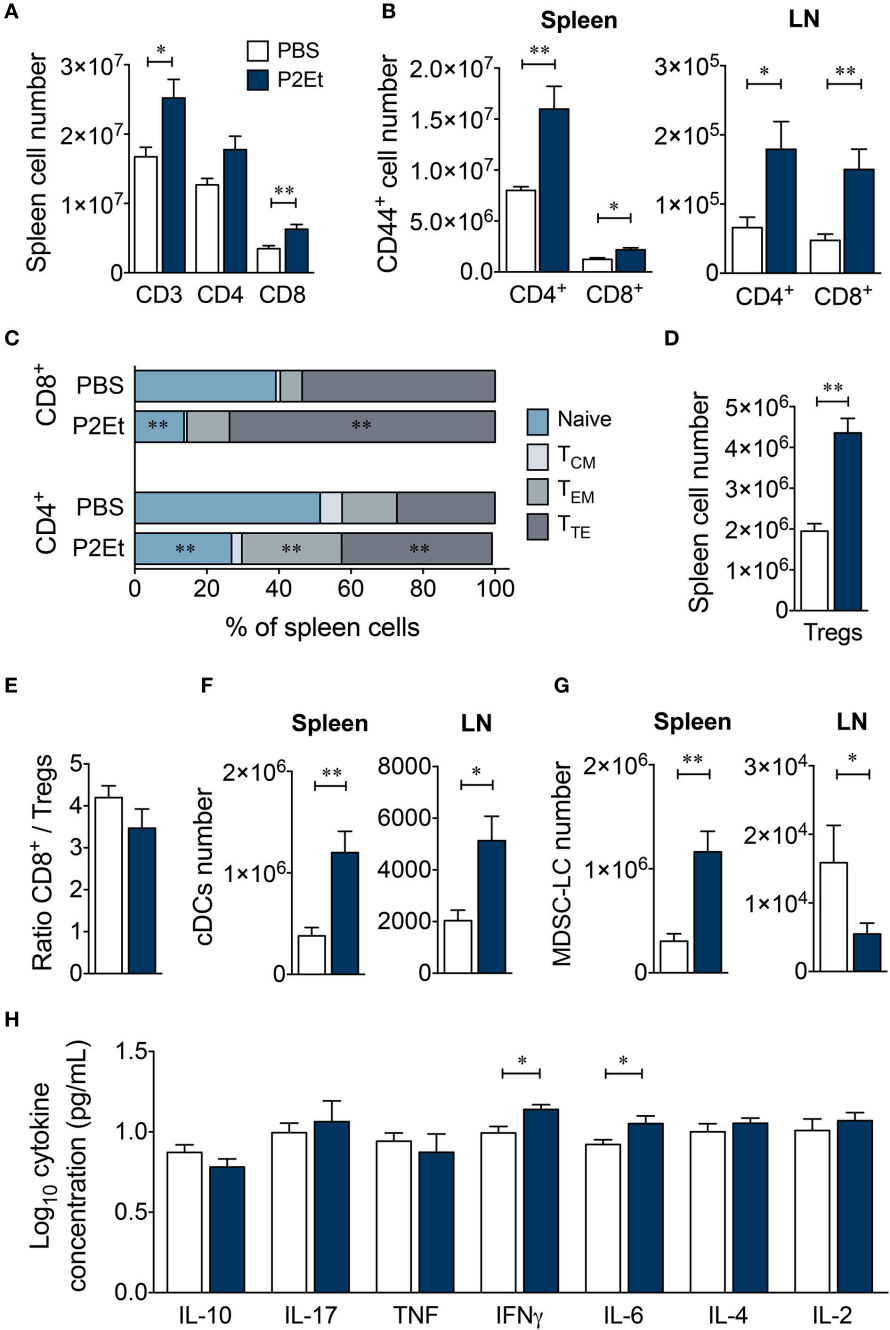
P2Et extract modulates the immune response in healthy BALB/c mice. **(A)** Absolute numbers of splenic CD3^+^, CD4^+^, and CD8^+^ T cells in healthy mice treated with P2Et extract or PBS (control). **(B)** CD44^+^ CD4^+^ and CD8^+^ T cells in spleen or LN. **(C)** Percentages of splenic CD4^+^ and CD8^+^ T cells with naive, T_CM_, T_EM_ and T_TE_ phenotypes. **(D)** Absolute numbers of splenic regulatory T cells. **(E)** Ratio of splenic CD8^+^/Treg cells. **(F)** Conventional DC numbers in spleen or LN in each mouse group. **(G)** Absolute numbers of MDSC-LCs in lymph nodes and spleen. **(H)** Cytokine concentrations in serum of PBS or P2Et-treated healthy BALB/c mice measured by CBA. In all cases, data are represented as the mean ± SEM. The *p*-values were calculated using a Mann-Whitney *U* test. *p < 0.05, **p < 0.01.

To study possible strain specificity for the response to P2Et, we also evaluated the effect of the extract in healthy BALB/c mice. P2Et induced significant increases in CD3^+^ and CD8^+^ T cell numbers and tended to increase the number of CD4^+^ T cells in spleen (Figure 6A). However, no differences were found in LN (data not shown). In addition, P2Et treatment induced higher numbers of activated CD44^+^ CD4^+^ and CD8^+^ T cells in the spleen and LN (Figure 6B), as well as effector CD4^+^ and CD8^+^ T cells in the spleen (Figure 6C). As observed for C57BL/6 mice, higher numbers of Tregs were found in the spleen from P2Et-treated mice (Figure 6D), but the CD8^+^/Treg ratio was not affected (Figure 6E). Conventional DCs were also increased in spleen and LN after P2Et treatment in comparison to the control group (Figure 6F); however, the MDSC-LC population increased in spleen but significantly decreased in LN (Figure 6G). In terms of cytokine production, mice treated with P2Et had only higher concentrations of IFN-γ and IL-6 in serum compared with C57BL/6, in which a more diverse profile was observed (Figure 6H). Taken together, these results showed that P2Et administration modulated the immune system already in tumor-free, healthy C57BL/6 and BALB/c mice, mainly at a systemic level, even if changes in the LN were only observed in the BALB/c strain. These differences could be explained by the genetic background and might be determinants of when the P2Et extract was used as a treatment. The finding that the P2Et extract has an immunomodulatory effect in C57BL/6 and BALB/c healthy mice suggests that P2Et extract administration, upon tumor engraftment, modifies the tumor microenvironment, making it more proinflammatory and/or immunosuppressive, which in turn may promote faster tumor growth.

### *In vivo* P2ET treatment delays tumor growth in the spontaneous Tyr::N-Ras melanoma model

**Figure 7:**
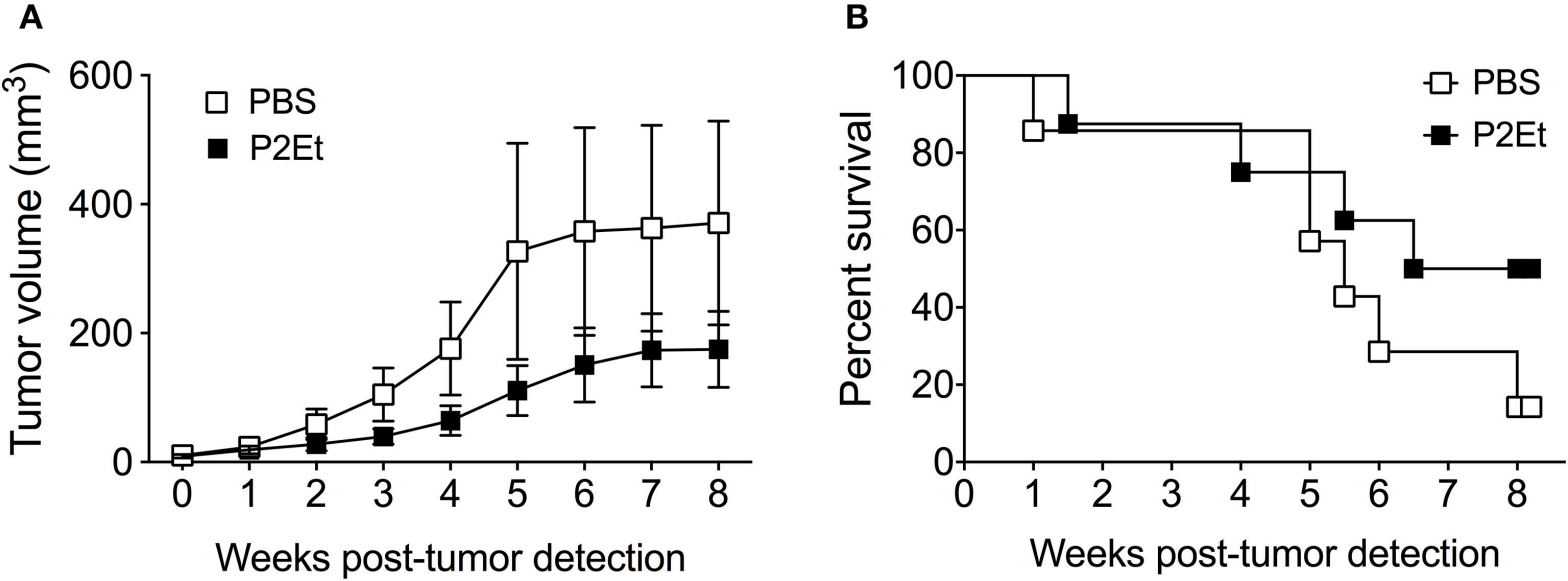
*In vivo* P2Et treatment delays tumor growth in the spontaneous Tyr::Nras melanoma model. **(A)** Tumor growth curve of Tyr:N-RasQ61K transgenic mice treated with PBS or P2Et. Data are represented as the mean ± SEM. **(B)** Kaplan-Meier survival curves of Tyr::N-Ras transgenic mice treated with PBS (*n* = 7) or P2Et (*n* = 8). The differences between groups in tumor growth and survival were not statistically significant.

Since P2Et administration induces activation of the immune system in healthy mice and can retard B16F10 tumor growth (16), and the prophylactic treatment seems to have a pro-tumoral rather than anti-tumoral effect, we wondered what would happen in the context of a spontaneous tumor model, a setting more closely resembling the clinical situation than transplantable tumor models. To address this question, we treated mice bearing spontaneous Tyr::N-Ras^Q61K^xINK4a-/- engineered melanomas, referred to thereafter as Tyr::Nras (23). The Tyr:N-Ras transgenic mice developed melanoma tumors 9 weeks after DMBA topic treatment. P2Et therapy initiated immediately after tumor detection was able to delay tumor growth (Figure 7A). In addition, P2Et-treated mice showed a trend toward higher survival rates compared with the littermate control mice (Figure 7B). These findings indicate that P2Et-therapy still exerts a protective role in spontaneous melanoma tumors at a stage when immune tolerance and tumor-escape likely occurred.

## Discussion

Polyphenols are widely present in food and beverages of plant origin. Numerous studies have focused on the antioxidant properties of polyphenols, but the beneficial effect of antioxidants *in vivo* remains controversial (28). Despite years of research, the value of antioxidants in the prevention and treatment of cancer is not yet clear, and it could not yet be determined whether antioxidants act as protective or pro-tumorigenic agents. These inconsistencies appear to be due to multiple factors, including the dose and type of antioxidant, route of administration and tumor type (29).

In this study, we not only confirmed our previous observations (16) and, additionally, observed a decrease in MDSC-LC after P2Et treatment, but we also showed that this immunostimulating effect was reversed when we used P2Et as a prophylactic treatment, apparently at the expense of a systemic pre-activation of the immune system that induced a proinflammatory environment. The interesting thing is that P2Et exerts an antioxidant effect at a basal level and after stressor treatment which does not change the cytokine profile produced by normal splenocytes of both BALB/c and C57BL/6 after stimulation with PMA/ionomycin (Supplementary Figures 1, 2). This means that the generation of a pro-inflammatory environment evidenced in the plasma of healthy animals, possibly depends on the complex interactions between the immune system and its microenvironment, which is different in animals with tumors, where this proinflammatory activity is increased. Likewise, it has been found that the P2Et extract has antioxidant activity in tumor cell lines such as B16 and K562 cells (unpublished data), however, this antioxidant activity does not protect them from apoptotic cell death (20), and combined treatment with P2Et and doxorubicin, of BALB/c mice ortotopically transplanted with breast cancer TSA cell line, significantly diminish tumor evolution compared with isolated treatment (19).

As recently reviewed, the protective effects of green tea, as well as its main polyphenol, the epigallocatechin gallate (EGCG), have been demonstrated in various studies, including (1) tumor development, as revealed in a 10-year prospective cohort study, (2) the prevention of colorectal adenoma recurrence, as observed in a double-blind randomized clinical phase II trial, (3) inhibition of metastasis of B16 melanoma cells to the lungs of mice, and (4) synergistic enhancement of anticancer activity against human cancer cell lines combining EGCG and anticancer compounds (30). Additionally, some polyphenols, such as resveratrol and curcumin, can modulate the immune response by decreasing TGFγ production and the accumulation of Tregs, thus restoring antitumor immunity (31, 32). Moreover, in aged rats, polyphenols derived from *Cassia auriculata* flowers have been shown to increase the frequencies of T and B cells and enhance splenocyte proliferation (33). Likewise, the extracts of ripe fruits from date palm increased the number of IFNγ^+^ CD4^+^ cells in the spleen of C3H/HeN mice (34), suggesting an immunostimulating effect of polyphenols.

In contrast to these data, in a non-tumoral context, polyphenols seem to have different functions. In fact, EGCG and a green tea extract containing 10 μM of EGCG induce IL-10 production and FoxP3 expression in Jurkat T cells *in vitro* and increase Treg frequencies *in vivo* in healthy BALB/c treated daily for 7 days (35). Interestingly, we also observed an increase in Tregs in both strains of healthy mice in response to P2Et treatment. However, the Treg population decreased when the animals were treated with P2Et in a tumoral context, but different results were obtained depending on the strain. These findings suggest that the regulation of FoxP3 expression mediated by polyphenols also depends on the genetic background.

Unexpectedly, pretreatment with P2Et favored tumor growth and metastasis in both BALB/c and C57BL/6 mice. This effect was related to a generalized decrease in CD4^+^ and CD8^+^ T cell frequencies in the lymphoid nodes and tumor (in both tumor models) and an increase in intratumoral MDSC-LCs compared with the controls, as well as a significant increase in IL-6 plasma levels in the animals.

Natural products, particularly tannic acid, have been implicated in the decrease in proliferation of normal lymphocytes and the production of IL-2 (36). However, a systematic review of the literature examining the effects of flavonoids on the immune response has shown that in the vast majority of studies, none of the parameters of the immune response evaluated in the different studies is affected in healthy individuals in response to prolonged consumption or large amounts of natural antioxidants. It is worth noting, however, that not many studies to date have evaluated the multi-parametric (cytokines, innate and adaptive immunity) roles of flavonoids in the modulation of the immune response. Interestingly, the systematic review shows that there was no effect on the reduction of TNF-α levels after intake of meals or flavonoid-rich supplements in any of the interventional studies conducted in healthy individuals (0/21). In contrast, in individuals at high risk of cardiovascular disease, flavonoids were found to decrease TNF-α levels in at least 30% of interventions (5/17). This effect was much more pronounced if the analysis was restricted to patients affected by different diseases, among which 60% of cases (4/7) showed an effective reduction of TNF-α values after supplementation with different mixtures enriched with polyphenols. Thus, it is possible that the influence of flavonoids on immunity is more effective in individuals for whom the presence of risk factors generated by the disease favors a greater challenge to the immune response, compared to individuals with a low degree of inflammation (37).

All the above findings raise the question of why preconditioning of the immune response with the P2Et extract favors tumor growth. It was recently found that the antioxidants N-acetylcysteine (NAC) and vitamin E increase the proliferation of human lung cancer cells and tumor growth in mice with B-RAF– and K-RAS–induced lung cancer by reducing ROS, DNA damage, and p53 (14). Additionally, administration of N-acetylcysteine (NAC) increased lymph node metastases in a spontaneous mouse model of malignant melanoma but had no impact on the number and size of primary tumors. Similarly, NAC increases the migration and invasive properties of human malignant melanoma cells without affecting their proliferation. This phenomenon is related to the synthesis of glutathione, which suggests that the relationship between antioxidants and glutathione play an important role in melanoma progression (15). However, it is possible that the antioxidant activity of polyphenols favor cellular proliferation by removing some of the tumor cells from a state of senescence induced by high levels of ROS, as described for some tumor models (38).

In contrast, some polyphenols, such as resveratrol, curcumin and EGCG, negatively regulate the expression of coactivator molecules. Particularly, resveratrol increases the expression of suppressor molecules, such as inhibitory immunoglobulin- including transcript −3 and −4 (ILT3, ILT4), and decreases the ability to produce proinflammatory IL-12 after activation, increasing the production of IL-10 upon stimulation with LPS. Interestingly, curcumin-treated DCs show impaired induction of Th1 responses (39). This suppression may promote a tolerogenic microenvironment prior to the appearance of a tumor, or in our model, prior to tumor transplantation, limiting the *in situ* inflammatory process, which is required for the generation of an effective immune response.

Another important element that should be considered in the use of polyphenols is the dose response effect. For example, resveratrol has a biphasic effect, where it increases the activity of cytotoxic T and NK cells at low concentrations and inhibits their activities at high concentrations (40, 41). Additionally, polyphenols seem to directly modulate CD4^+^ T cell activation and polarization possibly through modulation of the expression of CD28/CTLA-4. In fact, resveratrol and curcumin are able to decrease CD28 and CD80 expression and upregulate CTLA-4 and the IL-10 production (42). It is possible that the pretreatment with P2Et in our tumor models led to an accumulation of antioxidants, which finally decreased the immune response and favored the development of the tumor.

Another interesting result in the PBS/P2Et animals was the observed lower numbers of intratumoral MDSC-LCs than in the P2Et/P2Et-treated animals. The immunosuppressive activity of MDSCs on T lymphocytes (43–46) and NK cells (47, 48) and the polarization toward a tumor-promoting type 2 phenotype (49) has been widely documented. It has been reported that MDSCs can be targeted by antioxidants, which limits the *in vitro* expansion of MDSCs as well as their suppressive capacity (50, 51). However, MDSCs expand under inflammatory conditions (52); the latter would allow us to propose that the increase in proinflammatory cytokines in healthy animals, (Figure 5I) as well as the significant increase in IL-6 in tumor-bearing mice and mice pretreated with P2Et (Figure 4) could act in concert to favor the deviation of the immune response toward immunosuppression and be translated into MDSC proliferation and recruitment, as has been suggested in other models (53). The increase in IL-6 in animals pretreated with P2Et could, in fact, be a consequence of the pre-activation of the immune system and not a direct effect of P2Et, since we observed that IL-6 decreased in response to treatment with P2Et in the present work and in the 4T1 breast cancer model in a previous study (18). In fact, the role of polyphenols in the decrease of IL-6 production has been widely reported (42), particularly through inhibition of the NF-κB pathway involved in the synthesis of proinflammatory cytokines (54).

The differences between the two models regarding the specific response to the extract might be explained by the biological differences in the strains in terms of their inflammatory profile, as shown in other models (55). In addition, the biology of tumors is different. The B16-F10 tumor model is considered poorly metastatic, while the 4T1 breast cancer model is known to be highly metastatic to several distant organs, as confirmed by the larger number of metastatic foci at day 20 post-tumor implantation.

Analysis of the P2Et activity in the spontaneous Tyr:NRas model, the P2Et treatment started as soon as the tumors appeared. At this time, the initial control phase exerted by the immune response had failed, and the balance leaned thought the tumor growth. It is in this context that the treatment with P2Et improved the survival of the animals, possibly because the tumor microenvironment of them was already altered favoring the protective activity of P2Et. It would be convenient to evaluate if the previous treatment for several weeks before the tumor appears, is protective, and only in this way could we sustain its preventive capacity, however, the fact that it did not increase tumor growth is very favorable. It would also be appropriate to assess the redox state of the animal (or patient) before and after the onset of the disease, to determine when the break REDOX balance, plays in favor of the tumor growth or in favor of the immune response.

Our results are in agreement with the idea that polyphenols, particularly P2Et, can be used within the framework of anti-tumor therapy, but not in a prophylactic context. Further detailed studies are necessary to more precisely determine the role of antioxidants in the immune response and, above all, their protective or adjuvant role in the treatment of cancer. The advent of new technologies that allow multiparameter “omic” analysis involving the type of tumor, the genetic characteristics of patients, as well as their oxidation status and inflammatory profile, among others, are necessary to advance the development of new therapies based on the use of natural products.

## Author contributions

PL, AG-C, CU, AD, and AM-U: design and execution of experiments, acquisition, analysis and interpretation of data, and manuscript drafting. AB and PR: data interpretation and manuscript drafting. SF: leader of the project, design of the experiments, interpretation of the results, and manuscript revision. PL and SF: wrote the manuscript with contributions from all authors.

### Conflict of interest statement

SF and CU are inventors of a granted patent related to P2Et. The remaining authors declare that the research was conducted in the absence of any commercial or financial relationships that could be construed as a potential conflict of interest.
