# Supplementary material for: Prophylactic vs. Therapeutic Treatment With P2Et Polyphenol-Rich Extract Has Opposite Effects on Tumor Growth
Source: Front Oncol. 2018 Aug 30;8:356. doi: 10.3389/fonc.2018.00356 (PMC6127621; doi:10.3389/fonc.2018.00356)
Supplement: Supplementary file 1 [file Data_Sheet_1.docx]

Supplementary Material

Prophylactic versus Therapeutic Treatment with the P2Et Polyphenol-Rich Extract Has Opposite Effects on Tumor Growth

**Paola Lasso^1^, Alejandra Gomez-Cadena^1,2^, Claudia Urueña^1^, Alena Donda^2^, Amaia Martinez-Usatorre^2^, Alfonso Barreto^1^, Pedro Romero^2^, Susana Fiorentino^1*^**

*** Correspondence:**Dra. Susana Fiorentino
susana.fiorentino@javeriana.edu.co

# Supplementary Figures and Tables

## Supplementary Figures


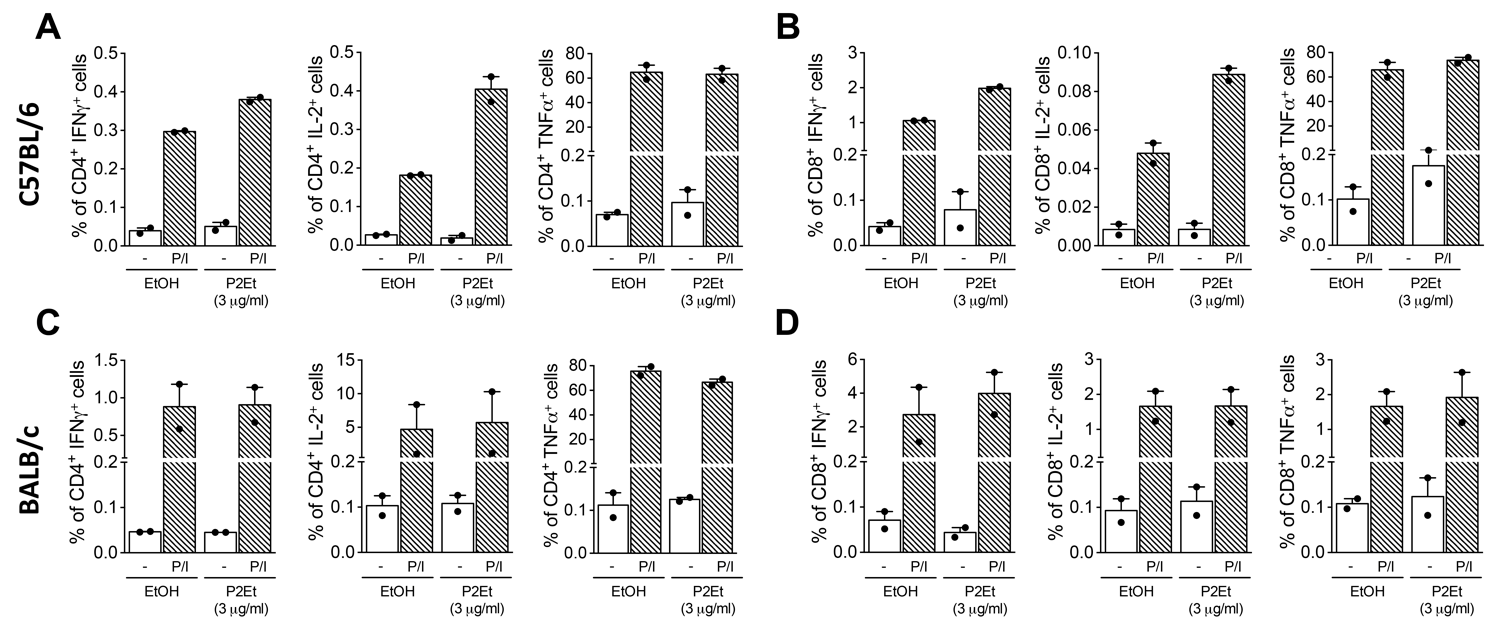


**Supplementary Figure 1.** ***In vitro* P2Et treatment dos not interfere with cytokine production in CD4^+^ and CD8^+^ T cells.** CD4^+^ **(A and C)** and CD8^+^ **(B and D)** T cells from spleen of two healthy C57BL/6 **(A and B)** or two healthy BALB/c **(C and D)** mice producing IFNγ, TNFα or IL-2 after 24 hours of *in vitro* P2Et or ethanol (EtOH) treatment with and without PMA/ionomycin (P/I) stimulation.

**Supplementary Figure 2. *In vitro* P2Et treatment has antioxidant effect over splenocytes from healthy mice.** P2Et antioxidant activity over splenocytes treated with 0, 1 and 10 μM of H_2_O_2_ from BALB/c **(A and B)** and C57BL/6 **(C and D)** healthy mice. Antioxidant activity was evaluated in cells treated with EtOH (control), Trolox (antioxidant control) and P2Et extract at 12 **(A and C)** and 24 hours **(B and D)** of culture.
